# Supplementary material for: An evaluation of the Live Well Erie workforce development program, jumping off the benefits cliff: A protocol paper
Source: PLoS One. 2024 Nov 14;19(11):e0311496. doi: 10.1371/journal.pone.0311496 (PMC11563387; doi:10.1371/journal.pone.0311496)
Supplement: Attachment — Submitted filename: PLOS ONE Response to Reviewers.docx [file pone.0311496.s001.docx]

Response to Reviewers

**1. When submitting your revision, we need you to address these additional requirements.**

We apologize for not adhering to the PLOS ONE style requirements during the initial submission. We have modified the manuscript to adhere to your publication criteria. We followed the style templates through the links you provided and modified the headings within the document and cited our references within brackets.

**2. PLOS requires an ORCID iD for the corresponding author in Editorial Manager on papers submitted after December 6th, 2016. Please ensure that you have an ORCID iD and that it is validated in Editorial Manager.**

The Orcid ID for the corresponding author has been included. [https://orcid.org/0000-0003-3501-2530](https://orcid.org/0000-0003-3501-2530?lang=en)

**3. We note that the grant information you provided in the ‘Funding Information’ and ‘Financial Disclosure’ sections do not match. When you resubmit, please ensure that you provide the correct grant numbers for the awards you received for your study in the ‘Funding Information’ section.**

The discrepancy between the information in the funding information and financial disclosure sections has been rectified. They now match and we have ensured that the grant number is indeed correct.

**4. Thank you for stating the following financial disclosure: 
 [Erie County Department of Social Services].  
Please state what role the funders took in the study.  If the funders had no role, please state: ""The funders had no role in study design, data collection and analysis, decision to publish, or preparation of the manuscript."" 
If this statement is not correct you must amend it as needed. 
Please include this amended Role of Funder statement in your cover letter; we will change the online submission form on your behalf.**

We have amended the statement in the cover letter as requested. The statement now reads, “The funders played no role in the design of the study protocol, however they made the final decision for publication and assisted in the preparation of the manuscript.”

**5. When completing the data availability statement of the submission form, you indicated that you will make your data available on acceptance. We strongly recommend all authors decide on a data sharing plan before acceptance, as the process can be lengthy and hold up publication timelines. Please note that, though access restrictions are acceptable now, your entire data will need to be made freely accessible if your manuscript is accepted for publication. This policy applies to all data except where public deposition would breach compliance with the protocol approved by your research ethics board. If you are unable to adhere to our open data policy, please kindly revise your statement to explain your reasoning and we will seek the editor's input on an exemption. Please be assured that, once you have provided your new statement, the assessment of your exemption will not hold up the peer review process.**

As this is a protocol paper, we don’t have complete data to share yet. However, we will be prepared to share de-identified quantitative data at the conclusion of the study.

**6. Please amend your list of authors on the manuscript to ensure that each author is linked to an affiliation. Authors’ affiliations should reflect the institution where the work was done (if authors moved subsequently, you can also list the new affiliation stating “current affiliation:….” as necessary).**

The list of authors has been amended to ensure each author is linked to an affiliation.

**7. Please amend either the abstract on the online submission form (via Edit Submission) or the abstract in the manuscript so that they are identical.**

The abstract on the online submission form and the manuscript now match.

**8. Please include your full ethics statement in the ‘Methods’ section of your manuscript file. In your statement, please include the full name of the IRB or ethics committee who approved or waived your study, as well as whether or not you obtained informed written or verbal consent. If consent was waived for your study, please include this information in your statement as well.**

The ethics statement has been included and inserted in the methods section of the manuscript file.

**9. Please review your reference list to ensure that it is complete and correct. If you have cited papers that have been retracted, please include the rationale for doing so in the manuscript text, or remove these references and replace them with relevant current references. Any changes to the reference list should be mentioned in the rebuttal letter that accompanies your revised manuscript. If you need to cite a retracted article, indicate the article’s retracted status in the References list and also include a citation and full reference for the retraction notice.**
The following references were included to address Reviewer #1 feedback.

**10. Reviewer #1: Study protocol clearly presented. Knowledge gaps identified. The authors might consider strengthening the literature review. There is an extensive literature on career pathways. More depth is needed.**

We express gratitude to the reviewer for their feedback. We incorporated the following statement on pg. 4, paragraph 2. “A more recent process evaluation of nine programs employing “career pathway” strategies for low-income and low-skilled adults, 18 months after random assignment into treatment and control groups, is showing promising results (Gardiner & Juras, 2019). While most of the programs being evaluated appear to be on-track to achieving their long-term goals, program participants are currently experiencing increases in average rate of employment and average earnings over successive follow-up quarters.” Additionally, we added the following sentence at the beginning of the paragraph, “A recent metanalysis of 46 impact evaluations of programs that utilize elements of the CP approach found that compared to control/comparison groups, CP program participants gain a 28-percentage point advantage in earning an educational credential, a 19-percentage point advantage in obtaining employment specific to the industry they train for, and small earnings gains in the short term ^[11].”^ The added sentences were drawn from the two citations cited below that have been added to the references.

1. Peck, L. R., Schwartz, D., Strawn, J., Weiss, C. C., Juras, R., Mills de la Rosa, S., Greenstein, N., Morris, T., Durham, G., & Lloyd, C. (2021). *A meta-analysis of 46 career pathways impact evaluations*. Abt Associates. <https://www.dol.gov/agencies/eta/research/publications/meta-analysis-46-career-pathways-impact-evaluations>
2. Gardiner, K. and R. Juras. (2019). PACE Cross-Program Implementation and Impact Study Findings, OPRE Report #2019-32, Washington, DC: Office of Planning, Research, and Evaluation, Administration for Children and Families, U.S. Department of Health and Human Services.
